# Intrawound vancomycin powder in orthopaedic surgery after the VPIP trial: a critical reappraisal of efficacy, dosing, application plane, and antimicrobial stewardship

**DOI:** 10.5194/jbji-11-343-2026

**Published:** 2026-06-12

**Authors:** Felix Werneburg, Alexander Zeh, Natalia Gutteck, Karl-Stefan Delank

**Affiliations:** 1 Department of Orthopaedic, Trauma and Reconstructive Surgery, University Hospital Halle (Saale), Martin Luther University Halle-Wittenberg, Ernst-Grube-Straße 40, 06120 Halle (Saale), Germany

## Abstract

Topical intrawound vancomycin powder has been widely adopted in orthopaedic surgery as an adjunct for the prevention of surgical site infection (SSI) and periprosthetic joint infection (PJI). Retrospective cohorts and derived meta-analyses have long suggested a consistent benefit, and the technique became established practice at many institutions. Over the past 5 years, prospective randomised evidence has added an important corrective to this picture. The VPIP trial (Saba et al., 2025) randomised 1901 high-risk patients undergoing primary hip or knee arthroplasty across 17 US centres and found no benefit of vancomycin, dilute povidone-iodine, or their combination over saline for any 3-month infection endpoint; a biostatistical projection to 80 000 patients left the number needed to treat near 500, and enrolment was closed for statistical futility. The VANCO trial in high-risk tibial fractures (O'Toole et al., 2021) narrowly missed its primary endpoint (P=0.06) but demonstrated a significant post hoc reduction in gram-positive deep infections without gram-negative shift. Mechanistic data indicate that sub-inhibitory vancomycin concentrations increase *Staphylococcus aureus* biofilm formation and raise infection rates in vivo – an effect not reproduced by cefazolin. Current international guidance does not issue a general recommendation for topical vancomycin, a position consistent with this more differentiated evidence base. The present review integrates these strands into an indication-specific framework that separates prophylactic application in clean surgical fields, where benefit is now in serious doubt, from adjunctive-therapeutic application in established infection, and identifies the settings in which topical vancomycin may still be considered individually and those in which it should be avoided.

## 1 Introduction

Surgical site infection and periprosthetic joint infection (PJI) rank among the most consequential complications of elective and trauma orthopaedic surgery. Reported PJI incidence after primary hip and knee arthroplasty ranges from 1 % to 2.3 %, rising above 5 % in high-risk populations, with an annual US economic burden projected to reach USD 1.9 billion by 2030 (Premkumar et al., 2021). In instrumented spinal surgery and in high-risk fracture fixation, infection rates can reach double-digit percentages in selected subgroups. Systemic perioperative antibiotic prophylaxis – typically a first- or second-generation cephalosporin – is the established cornerstone of prevention across international guidelines (WHO, 2018; Berríos-Torres et al., 2017); its efficacy is well documented, and the margin for incremental improvement is limited.

Against this background, the intraoperative application of crystalline antibiotic powder to the surgical wound has an older pedigree than is often appreciated: topical vancomycin was first described in cardiac surgery, where Vander Salm et al. (1989) applied the powder to the cut sternal edges to prevent deep sternal wound infection. Adapted to orthopaedics and popularised in instrumented spinal fusion by Sweet et al. (2011), application of vancomycin powder directly before wound closure emerged in the early 2010s as a simple, inexpensive, and pharmacologically plausible adjunct. Local wound concentrations several orders of magnitude above the minimum inhibitory concentration (MIC) for methicillin-resistant *Staphylococcus aureus* (MRSA) are readily achievable, while systemic absorption remains negligible. Numerous retrospective cohorts and derived meta-analyses reported reductions in postoperative infection rates, particularly in instrumented spinal surgery and primary arthroplasty (Chiang et al., 2014; Liao et al., 2022; Shan et al., 2020), and within a decade topical vancomycin had become established practice at many institutions, endorsed by the North American Spine Society as an adjunctive low-cost strategy (Shaffer et al., 2013). It is worth stating at the outset what the review does and does not encompass: crystalline powder is only one – and, in absolute terms, a minority – form of local antibiotic delivery, distinct from the carrier-based modalities (e.g., antibiotic-loaded bone cement, articulating and static spacers, bioactive glass, and resorbable calcium sulfate) that serve principally as therapeutic depots and dead-space management in established infection. We confine the discussion to crystalline powder but consider deliberately both its prophylactic use in clean fields and its adjunctive-therapeutic use in the presence of an identified pathogen.

The evidence base has since matured. In high-risk tibial fractures, the VANCO trial narrowly missed its primary endpoint but demonstrated a selective post hoc reduction in gram-positive deep infection (O'Toole et al., 2021). In primary hip and knee arthroplasty, the VPIP trial showed no advantage of topical vancomycin, dilute povidone-iodine (DPI), or their combination over saline and was closed for statistical futility after 1901 patients (Saba et al., 2025). Mechanistic work has added a counter-intuitive pharmacodynamic consideration: sub-inhibitory vancomycin concentrations promote *S. aureus* biofilm growth and raise infection rates in vivo, an effect not observed with cefazolin (Brothers et al., 2023). Current international guidance is accordingly cautious – major bodies, including the WHO and CDC, and recent European national guidelines do not issue a general recommendation for topical vancomycin – and the stewardship implications of broad glycopeptide exposure further shape the current clinical debate, given that vancomycin remains a glycopeptide of high clinical importance and its topical use is off-label.

This review reappraises intrawound vancomycin powder in light of these developments. Section 3 integrates the pivotal trials and the most recent trial-sequential meta-analytic evidence by indication. Section 4 examines dosing and the sub-MIC biofilm paradox. Section 5 addresses the subfascial versus suprafascial application question. Section 6 synthesises the safety profile. Section 7 reviews the stewardship framework across international guidelines. Section 8 translates the evidence into an indication-specific recommendation matrix that separates prophylactic from adjunctive-therapeutic use.

## 2 Methods

This is a structured narrative review rather than a systematic review or meta-analysis. Searches of PubMed/MEDLINE, Embase, Cochrane Central, and Web of Science were conducted through March 2026 using the terms “vancomycin powder”, “topical vancomycin”, “intrawound vancomycin”, “surgical site infection”, “periprosthetic joint infection”, “VPIP trial”, and “VANCO trial”. Randomised trials, cohort studies, meta-analyses, pharmacokinetic and mechanistic studies, and relevant international and national clinical guidelines were considered. Non-English sources were included where central to the argument. Evidence was appraised with regard to design, risk of bias, external validity, and stewardship implications. Recommendations were developed by author consensus.

## 3 Clinical efficacy: a critical appraisal

Two large randomised trials – VANCO in trauma surgery (O'Toole et al., 2021) and VPIP in primary arthroplasty (Saba et al., 2025) – have substantively refined the evidence base, and the trial-sequential analysis of Saka et al. (2024) has clarified the limits of the current randomised evidence across major orthopaedic surgery. Table 1 summarises these pivotal studies and should be consulted alongside the discussion by indication that follows.

**Table 1:** Pivotal studies driving the current evidence base for intrawound vancomycin powder in orthopaedic surgery.

Study	Design	Population and intervention	Primary finding	Interpretation
Saba et al. (2025) VPIP trial	Four-arm multicentre RCT; 17 US centres	1901 high-risk THA/TKA; 2 g vancomycin vs. 0.35 % DPI vs. combination vs. saline	No significant differences; projection to 80 000 patients leaves NNT ≈ 500; closed for futility	No prophylactic benefit detected in primary arthroplasty; not a formal non-inferiority proof
O'Toole et al. (2021) VANCO trial	Multicentre RCT; 36 US trauma centres	980 high-risk tibial plateau/pilon fractures; 1 g vancomycin vs. standard care	Deep SSI 6.0 % vs. 9.2 % (P=0.06); post hoc gram-positive reduction 6.8 % → 3.3 %	Selective benefit against gram-positive deep infection in high-risk fracture care
Saka et al. (2024)	RCT meta-analysis with trial sequential analysis	8 RCTs; 4307 participants; major orthopaedic surgery	Required information size 19 233 not reached; only gram-positive SSI significant	Current RCT evidence insufficient to confirm or reject a clinically relevant effect
Liao et al. (2022)	Dose-stratified meta-analysis	14 studies; 35 418 participants; hip and knee arthroplasty	Revision TKA RR 0.33; revision THA RR 0.37; inconsistent 2 g effect in primary THA	Supports adjunctive use in revision arthroplasty; context-specific interpretation in primary arthroplasty after VPIP
Brothers et al. (2023)	In vitro biofilm assays + murine abscess model	MRSA JE2; MSSA Newman; MSSA SH1000 at $\frac{1}{4}$ and $\frac{1}{2}$ MIC vancomycin	Biofilm formation ↑ 1.3–2.4-fold; in vivo infection rate 41.5 % → 62.5 % (P=0.03)	Sub-MIC exposure is substance specifically biofilm-promoting; under-dosing is not neutral

DPI, dilute povidone-iodine; MIC, minimum inhibitory concentration; NNT, number needed to treat; RCT, randomised controlled trial; RR, risk ratio; SSI, surgical site infection; THA, total hip arthroplasty; TKA, total knee arthroplasty.

### 3.1 Spinal surgery

Instrumented spinal surgery saw the earliest and broadest clinical uptake, and in routine practice the powder is rarely applied indiscriminately: its use tends to concentrate in selected high-risk strata – revision and long instrumented constructs; multilevel and deformity fusions; and patients with substantial comorbidity, diabetes, prior infection, or prolonged operative time. The seminal cohort of Sweet et al. (2011) reported a reduction in deep SSI from 2.6 % to 0.2 % in 1732 thoracolumbar fusions, a signal reinforced by several subsequent single-centre cohorts (O'Neill et al., 2011; Molinari et al., 2012; Strom et al., 2013; Caroom et al., 2013). The randomised evidence has proved less consistent. Tubaki et al. (2013) found no significant difference in 907 patients (1.61 % vs. 1.68 %), and Salimi et al. (2022) confirmed the neutral finding in 375 patients with a concerning signal toward gram-negative shift. A high-risk cohort in southern Türkiye documented a reduction from 6.54 % to 1.96 % (Oktay et al., 2021), but the retrospective design limits causal inference.

The methodologically most rigorous synthesis is the trial-sequential meta-analysis by Saka et al. (2024), which pooled eight RCTs across major orthopaedic surgery (4307 participants) and calculated a required information size of 19 233 patients. The cumulative Z curve crosses neither the signalling nor the futility boundary; only the subgroup of gram-positive SSI reached significance (RR 0.52; 95 % CI 0.32–0.83), with very low GRADE certainty. Shan et al. (2020) illustrate the same fundamental issue: the overall pooled estimate across 31 studies is RR 0.39, whereas the RCT-only sensitivity analysis yields a non-significant RR of 1.22. A structural limitation underlies these figures and is easily overlooked: the randomised evidence is generated almost entirely in unselected or mixed-risk cohorts and is not stratified by the very risk factors that govern selective use in practice. The pivotal question – whether a benefit undetectable across an unselected population is nonetheless concentrated within a definable high-risk stratum – cannot therefore be resolved from the current data and is more accurately characterised as an evidence gap than as demonstrated inefficacy. This divergence between retrospective and prospective evidence is a recurring feature of the field and is examined in Sect. 3.5.

### 3.2 Primary and revision arthroplasty

Retrospective evidence in arthroplasty is predominantly positive. Matziolis et al. (2020) reported a reduction in TKA infection rates from 1.3 % to 0.3 % (P=0.033) with 1 g of intra-articular vancomycin powder; the parallel THA reduction from 1.1 % to 0.5 % did not reach significance. Dial et al. (2018) documented a reduction from 5.5 % to 0.7 % in 265 THA patients but at the cost of 4.4 % sterile-wound complications in the intervention arm versus 0 % in controls. Meta-analyses of mixed retrospective and prospective data yielded pooled odds ratios of around 0.41 (Peng et al., 2021) – estimates that require reassessment in light of the randomised evidence discussed below.

In the randomised setting, a different picture emerges. The VPIP trial (Saba et al., 2025) randomised 1901 high-risk patients – 821 THA and 1080 TKA – at 17 US centres to four arms: 2 g vancomycin powder alone (1 g subfascial plus 1 g suprafascial), 0.35 % DPI lavage alone, combined vancomycin plus DPI, or saline irrigation. No significant differences between arms emerged for any infection-related endpoint (3-month infection rate P=0.14 for THA; P=0.13 for TKA; wound complications 3.2 % and 2.9 %, respectively). Two clarifications by the investigators are important for interpretation. First, the originally planned non-inferiority design (target n=13400) was abandoned after biostatistical consultation because formal non-inferiority testing at this low event rate would have required prohibitively large cohorts. Analysis proceeded by chi-square comparisons and therefore does not constitute a formal non-inferiority proof. Second, the ad hoc analysis at n=1901 showed that extending enrolment to 80 000 patients would not yield meaningful infection trends and that the number needed to treat would remain near 500 for septic reoperation and 250 for any infection requiring reoperation. On these grounds, enrolment was closed for statistical futility, and the investigators place the use of prophylactic vancomycin or DPI in high-risk primary arthroplasty at the discretion of the surgeon or institution.

The situation in revision arthroplasty differs. The dose-stratified meta-analysis by Liao et al. (2022; 14 studies, 35 418 participants) reports significant effects for revision procedures (RR 0.33 (95 % CI 0.14–0.77) for RTKA; RR 0.37 (0.14–0.96) for RTHA), while acknowledging the narrow evidence base and inconsistencies in the 2 g subgroup for primary THA. Gao et al. (2024; 22 studies, 23 363 participants, comprising 19 retrospective cohorts and 3 RCTs) make the methodological problem explicit: the retrospective studies yield a pooled RR of 0.36 (95 % CI 0.22–0.59), whereas the three available RCTs yield a non-significant RR of 0.39 (95 % CI 0.02–6.78; P=0.52). The distinction between primary prophylactic and septic adjunctive-therapeutic use therefore has real evidentiary consequences.

### 3.3 Orthopaedic trauma

The VANCO trial (O'Toole et al., 2021) randomised 980 patients in modified intention-to-treat analysis – 481 intervention, 499 control – with operatively treated tibial plateau or pilon fractures and at least one high-risk feature (open fracture, compartment syndrome, staged fixation, or NNIS risk index ≥2) across 36 US trauma centres. A single dose of 1000 mg vancomycin powder was applied over the internal fixation construct before closure. The primary endpoint of deep SSI within 182 d narrowly missed the conventional significance threshold: complete case 6.0 % vs. 9.2 %, Kaplan–Meier 6.4 % vs. 9.8 %, and risk difference -3.4 percentage points (P=0.06). The post hoc analysis revealed a significant reduction in gram-positive deep infections from 6.8 % to 3.3 %, while gram-negative deep infections remained numerically comparable (11 vs. 10 cases) – no evidence of a compensatory pathogen shift. The secondary analysis by Joshi et al. (2024) confirmed the selective reduction in gram-positive cocci infections (3.7 % vs. 8.0 %; P=0.01) without a compensatory resistance shift; notably, this reduction was driven predominantly by methicillin-susceptible *S. aureus*, the incidence of MRSA infection being comparable between arms, so that a specifically MRSA-directed benefit cannot be inferred from the trial. The VANCO investigators interpret these findings cautiously: not all pre-specified analyses reached the 5 % threshold, but the signal supports a clinically meaningful reduction in gram-positive deep infection in high-risk fracture populations.

### 3.4 Septic revision and soft-tissue infection

Septic revision surgery occupies a conceptually distinct position: here, vancomycin powder is applied in the presence of an identified pathogen as part of a comprehensive infection treatment strategy, not as prophylaxis in a clean field. The pharmacodynamic rationale differs accordingly. Biofilm-eroding local concentrations that cannot be achieved by systemic administration without prohibitive toxicity are delivered directly to the site of infection. This adjunctive-therapeutic role aligns with the pharmacodynamics of glycopeptides, whose efficacy depends on time above MIC and local penetration into sessile bacterial populations.

Randomised evidence specific to septic revision is limited. The Liao et al. (2022) meta-analysis provides the most directly relevant synthesis and reports significant reductions in PJI recurrence with adjunctive vancomycin powder in both revision TKA (RR 0.33; 95 % CI 0.14–0.77) and revision THA (RR 0.37; 95 % CI 0.14–0.96). These estimates derive from a small number of predominantly retrospective studies and must be interpreted with caution, but they converge with the pharmacodynamic rationale and with consistent signals from case series using 1000–2000 mg in two-stage exchange arthroplasty. In infected nonunion and chronic osteomyelitis, the same logic applies – local high-concentration delivery as part of multidisciplinary infection management, rather than prophylaxis in isolation. The absence of definitive randomised evidence in these settings should not be read as equivalence to the primary-prophylactic scenario because the therapeutic intent, the microbiological target, and the risk–benefit calculus are fundamentally different. Stewardship principles favour the adjunctive-therapeutic application over routine prophylaxis in clean fields (Sect. 7.3), and this distinction is the conceptual keystone of the recommendation matrix in Sect. 8.

### 3.5 Why randomised and meta-analytic evidence diverges

The divergence between positive retrospective cohorts and neutral-to-negative randomised evidence has well-documented methodological causes (Mancino et al., 2023): study heterogeneity, pre–post designs with uncontrolled co-interventions (antiseptic skin preparation, drain management, glycaemic control), publication bias, and surveillance bias. The trial-sequential analysis of Saka et al. (2024) additionally demonstrates that the current randomised evidence lacks the information size to confirm or reject a small-to-moderate effect, and variability in outcome definitions – superficial versus deep SSI, MSIS, or ICM criteria for PJI – compounds the problem. A further, rarely stated observation points the same way: with the singular exception of the VPIP projection (a number needed to treat near 500 for septic reoperation and near 250 for any infection requiring reoperation; Saba et al., 2025), the literature does not report how many patients must be treated to prevent one infection – an omission that is itself instructive, since a preventive measure whose number needed to treat plausibly lies in the hundreds is hard to justify for routine, indication-independent use. A uniform recommendation across all indications is therefore difficult to defend on the current evidence; differentiation by indication is the more rigorous response.

## 4 Dosing and sub-inhibitory concentrations

No regulatory product information exists for topical application. Dosing is based on clinical experience and ranges in the literature from 500 to 4000 mg, with a single dose of 1000 mg representing the de facto standard derived from the spinal literature. Two pharmacodynamic considerations – dose-comparison evidence and the sub-MIC biofilm paradox – deserve separate treatment.

### 4.1 Dose-comparison studies and pharmacokinetics

Direct dose-comparison studies remain rare. Kunakornsawat et al. (2019) compared 1 g with 2 g in instrumented spondylodesis and found comparable SSI rates but a higher rate of sterile-wound secretion in the 2 g arm. The dose-stratified meta-analysis by Liao et al. (2022) supports significant effects at 1 g for both TKA (RR 0.38) and THA (RR 0.37), while 2 g showed no benefit in primary THA (RR 1.02). The systemic pharmacokinetics of topical application are well characterised: serum concentrations remain predominantly below the detection limit or well below the nephrotoxic threshold (Sweet et al., 2011; Schär et al., 2021), a finding corroborated by the absence of clinically relevant renal function decline in our institutional elective cohort (Beschauner et al., 2025). The systemic safety margin therefore permits doses up to 2000 mg in larger wound cavities without anticipation of systemic toxicity. The procedural footprint of the technique is minimal – distributing the powder before closure adds well under 2 min, and to our knowledge no trial has reported added operative time as a formal outcome – and the agent itself is inexpensive, at a few euros per gram. Low unit cost should not, however, be mistaken for cost-effectiveness: no formal economic evaluation incorporating the VPIP futility data has been published, and a number needed to treat nearly 500 in primary arthroplasty renders a favourable prophylactic cost-effectiveness ratio in that indication implausible, irrespective of the price per dose.

### 4.2 The sub-MIC biofilm paradox

**Figure 1:**
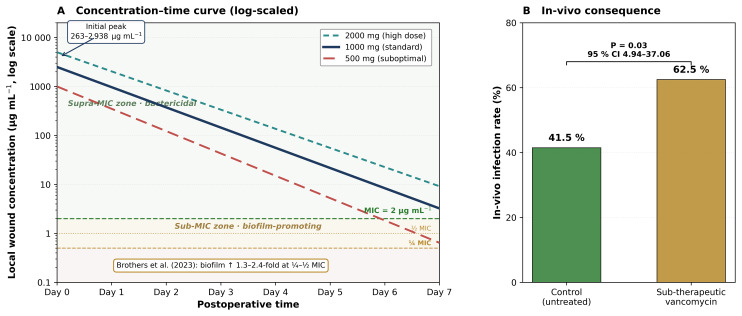
Conceptual local vancomycin concentration-time curve after intraoperative topical powder application. **(A)** Log-scale concentration–time relationship for 500, 1000, and 2000 mg doses. The minimum inhibitory concentration (MIC, 2 µg mL^−1^), $\frac{1}{2}$ MIC (1 µg mL^−1^), and $\frac{1}{4}$ MIC (0.5 µg mL^−1^) are indicated by horizontal reference lines (dashed for MIC and $\frac{1}{4}$ MIC; dotted for $\frac{1}{2}$ MIC). Initial wound concentrations reach 263–2938 µg mL^−1^ and traverse the sub-MIC range over the following days in a dose-dependent manner – most rapidly after 500 mg, later and more gradually after 1000 and 2000 mg; the sub-MIC transitional band corresponds to the biofilm-promoting range identified by Brothers et al. (2023). **(B)** In vivo consequence in the murine subcutaneous abscess model – infection rate of 41.5 % in untreated controls versus 62.5 % after sub-therapeutic vancomycin pre-exposure (P=0.03; 95 % CI 4.94–37.06; Brothers et al., 2023). Schematic representation; not to scale.

Brothers et al. (2023) examined the pharmacodynamic consequences of sub-inhibitory vancomycin exposure in planktonic growth assays, biofilm models (titanium rods and fibrinogen-coated plates), and a murine subcutaneous abscess model across three *S. aureus* strains – MRSA USA300 JE2, MSSA Newman, and MSSA SH1000. At concentrations of $\frac{1}{4}$ MIC (0.25 µg mL^−1^) and $\frac{1}{2}$ MIC (0.5 µg mL^−1^), biofilm formation was elevated 1.3- to 2.4-fold relative to untreated controls across all three strains. In the murine model, sub-therapeutic vancomycin pre-exposure raised the infection rate from 41.5 % to 62.5 % (P=0.03; 95 % CI 4.94–37.06; Fig. 1). Cefazolin did not reproduce this effect at any sub-MIC concentration – the finding is substance-specific, not a general sub-inhibitory phenomenon. The pathophysiological basis is partly clarified by Doroshenko et al. (2014): sub-inhibitory pre-exposure enhances extracellular DNA release, which further restricts antibiotic diffusion into the biofilm.

For topical powder application, the implications are direct. Local wound concentrations immediately after application reach 263–2938 µg mL^−1^ – up to approximately 1000-fold the MIC against MRSA – and subsequently decay in a dose-dependent manner, with standard 1000 mg doses typically falling below the MIC within 3–4 d (Fig. 1A). During this decay phase, the wound milieu inevitably traverses the sub-MIC range, with the consequences described above. When the initial concentration is not raised well above the MIC, the pro-biofilm-forming phase is prolonged relative to the short bactericidal initial phase. A clinically relevant corollary follows: in topical application, under-dosing is potentially more harmful than no application at all. The wide variability in published dose recommendations (500–4000 mg) is, on this reading, a methodological concern in its own right.

### 4.3 Sterile-wound complications and cytotoxicity

At the opposite end of the dose spectrum, sterile-wound complications define the most relevant local safety limit. Dial et al. (2018) observed sterile-wound complications in 4.4 % of intervention cases versus 0 % of controls in primary THA. In vitro data consistently indicate dose-dependent cytotoxicity at high local concentrations: Rathbone et al. (2011) demonstrated impaired osteogenic cell viability and matrix production above 1000 µg mL^−1^, and Röhner et al. (2021) reported chondrotoxicity at clinically achievable local concentrations. Two practical points frame the dosing decision. First, crystalline powder is a surface-applied adjunct rather than a dead-space management material: it provides no structural fill and elutes within a few days so that where antimicrobial management of dead space is genuinely required – large defect cavities and infected nonunion – carrier-based systems (antibiotic-loaded calcium sulfate, cement beads, bioactive glass) are the appropriate modality and fall outside the scope of this review. Second, the considerations of Sect. 4.1–4.3 converge on a pragmatic dosing logic: 1000 mg as the standard single dose; escalation to 2000 mg reserved for large revision cavities with high dead space and an adjunctive-therapeutic intent; avoidance of doses below 500 mg in larger wounds, where premature sub-MIC decay risks the biofilm-promoting effect (Fig. 1A); and explicit account of wound drainage, which accelerates elution and shortens the supra-MIC window.

## 5 Application plane: subfascial versus suprafascial

The anatomical plane of application is among the least systematically studied aspects of the technique. Terminology is inconsistent: subfascial denotes placement beneath the deep fascia in direct contact with muscle, bone, implant, or neural structures; suprafascial denotes placement in the subcutaneous layer above the deep fascia. The pharmacodynamic rationale differs between the two. Subfascial application targets the deep compartment where biofilm-mediated persistence occurs but entails direct exposure of osteogenic and chondrogenic cells to high concentrations. Suprafascial application minimises such contact but may leave the infection-relevant deep compartment sub-therapeutically exposed.

Only one direct comparative clinical study exists. Haimoto et al. (2018) compared subfascial with suprafascial application in a retrospective spinal cohort and reported comparable SSI rates with numerically fewer local adverse events in the suprafascial group; the study is limited by non-randomised design and modest sample size. Schär et al. (2021) documented negligible systemic absorption after suprafascial application in a pilot of the Swiss VANCO spinal trial, corroborating the systemic safety of this approach. The current evidence does not support a strict preference. A pragmatic indication logic approach is appropriate – subfascial placement in instrumented procedures with direct implant or bone exposure and suprafascial placement in situations with a high soft-tissue infection risk – with the default of 1000 mg distributed between planes where anatomical conditions permit.

## 6 Safety profile

Several years of clinical experience have established the safety profile of topical vancomycin as among the better-characterised aspects of the technique. Two domains warrant separate consideration: systemic pharmacotoxicity and local wound safety, including the hypothesis-generating aseptic signal from VPIP.

### 6.1 Systemic safety

The nephrotoxicity of systemic vancomycin has been recognised since 1958; reported incidences of vancomycin-associated acute kidney injury range from approximately 5 % in elective cohorts to above 40 % in intensive care populations (Yasrebi-de Kom et al., 2025), and concomitant piperacillin/tazobactam amplifies this risk (Pan et al., 2025). After topical application, the picture is consistently reassuring: Sweet et al. (2011), O'Toole et al. (2021), and Beschauner et al. (2025) report predominantly non-detectable serum concentrations and no clinically meaningful decline in renal function. In the Halle elective series of 50 patients, only two cases of KDIGO stage 1 acute kidney injury were observed, both resolving without sequelae. Systemic hypersensitivity phenomena are correspondingly exceptional after topical use: red-man syndrome is a rate-dependent, non-allergic reaction tied to systemic vancomycin exposure and is essentially not expected after powder application given negligible absorption, while severe delayed reactions are likewise a concern of systemic rather than topical therapy. Consistent with this, Ghobrial et al. (2015) recorded only 23 complications across 9721 spinal cases (0.3 %). Systemic toxicity is therefore not the limiting factor within standard dose ranges.

### 6.2 Local wound safety and the VPIP aseptic signal

Sterile-wound complications – discussed in Sect. 4.3 in the dosing context – represent the principal local safety limit. A further hypothesis-generating signal emerged from the VPIP trial: aseptic revision rates in the THA cohort were 3.0 % in the vancomycin-only arm, 1.9 % in the DPI-only arm, 0 % in the combination arm, and 1.1 % in the saline control (overall P=0.07). The VPIP investigators considered a vancomycin-crystal-related wear mechanism but discounted it with reference to the in vitro wear simulation of Qadir et al. (2014), in which 10 million loading cycles produced no differences between UHMW-polyethylene and cobalt–chromium couplings with or without vancomycin. The signal remains unexplained. While it does not, on its own, mandate a change in practice, it warrants systematic attention in future long-term analyses.

## 7 Antimicrobial stewardship and the international guideline framework

The use of a glycopeptide of high clinical importance in prophylaxis engages the core principles of antimicrobial stewardship (AMS): the frame of reference shifts from the individual patient to the population, and the relevant question becomes what broad glycopeptide exposure means for resistance emergence, pathogen selection, and the long-term therapeutic availability of vancomycin.

### 7.1 International guideline positions

Major international bodies have reached broadly convergent positions. The 2018 WHO Global Guidelines for the Prevention of Surgical Site Infection advise against the use of topical vancomycin powder (conditional recommendation, low-quality evidence; WHO, 2018), and the 2017 CDC SSI guideline (Berríos-Torres et al., 2017) makes no positive recommendation for topical antimicrobial application. Both were issued before the major randomised trials, and their cautious stance has since been reinforced by the VPIP and VANCO results. The North American Spine Society guideline (Shaffer et al., 2013) endorsed topical vancomycin as a low-cost adjunct in spinal surgery; this position predates the current randomised evidence and has not been formally updated. The German S3 guideline on perioperative antibiotic prophylaxis (AWMF 067-009; DGHM, 2025) is the most recent European assessment and concludes, after review of both the randomised and the meta-analytic evidence, that no general recommendation can currently be issued for intrawound vancomycin in spinal surgery – a position reasoned on discrepant study data, rising resistance rates, the lack of gram-negative coverage, and signals of pathogen shift (Ghobrial et al., 2014; 60.7 % gram-negative cultures in the vancomycin group versus 21 % in controls). The dedicated musculoskeletal-infection consensus bodies are consistent with this caution: the International Consensus Meeting on Musculoskeletal Infection, whose diagnostic criteria were used to define PJI in the VPIP trial, addressed local antibiotic delivery in its proceedings (Anemüller et al., 2019) without endorsing routine intrawound vancomycin as a standard of care, and to our knowledge no recommendation specific to vancomycin powder has been issued by EBJIS.

### 7.2 Pathogen shift and resistance

The meta-analytic evidence on pathogen shift under topical glycopeptide exposure is inconsistent. Gande et al. (2019; 28 studies, 19 302 patients) reported a reduction in gram-positive SSI from 70 % to 45.1 % (P<0.05) and a concomitant rise in gram-negative and polymicrobial SSI from 18.5 % to 35.8 % (P<0.05); the relative risk of gram-negative or polymicrobial SSI was elevated by 93.5 %. By contrast, the VANCO secondary analysis (Joshi et al., 2024) found selective reduction in gram-positive cocci (3.7 % vs. 8.0 %; P=0.01) without a compensatory gram-negative increase. The evidence for pathogen shift is therefore not conclusive, but it is sufficient to justify a cautious stewardship stance, particularly in broad indication-independent use. The concern is sharpened by a consideration of spectrum: an agent that extends only gram-positive cover can, by construction, address only that part of the eventual SSI spectrum left uncovered by standard cephalosporin prophylaxis, and where the breakthrough spectrum is thereby displaced toward gram-negative or polymicrobial pathogens – a risk most acute in the lower lumbar and sacral region given proximity to perineal and enteric flora – the empirical treatment of any subsequent infection may demand broader-spectrum agents, compounding rather than relieving selection pressure. No study to our knowledge has directly quantified subsequent broad-spectrum antibiotic consumption after intrawound vancomycin, which is a concrete target for stewardship-oriented research (Sect. 9). Evidence on specific vancomycin resistance is more reassuring: Chotai et al. (2017) studied 2802 patients and found no rise in vancomycin-resistant organisms, though a shift toward gram-negative pathogens and culture-negative seromas was evident. Neither the FDA nor the EMA has approved vancomycin for topical intraoperative wound application; such use is therefore off-label across major regulatory jurisdictions, with implications for patient information, documentation, and institutional governance.

### 7.3 Prophylaxis versus adjunctive therapy

The conceptual distinction between prophylactic use in the absence of proven infection and adjunctive-therapeutic use in the context of established infection – an infected prosthesis, an infected nonunion, and chronic osteomyelitis – is central from an AMS perspective, and it disposes of a common misconception: intrawound vancomycin powder is not itself an instrument of stewardship. In current practice it is invariably given in addition to, never instead of, guideline-concordant systemic prophylaxis or therapy, so it cannot produce the antibiotic-sparing effect that defines a stewardship intervention; the only route by which it might reduce downstream systemic antibiotic use – a consistent, clinically relevant fall in overall SSI or PJI incidence – is precisely what the randomised evidence fails to demonstrate; and its broad, undirected anti-gram-positive activity is conceptually at odds with targeted stewardship. The bearing of stewardship on this technique is therefore as a constraint on use, not as an argument for it. Adjunctive-therapeutic application against a defined pathogen is, by the same logic, more compatible with stewardship principles than routine prophylaxis in non-infected fields, especially in indications where randomised evidence of prophylactic benefit is absent. Two implications follow: topical application must be understood as additive to, not a substitute for, guideline-concordant systemic prophylaxis, and indications should be tied to actual infection risk, not to institutional habit. These principles translate directly into the recommendation matrix of Sect. 8.

## 8 Indication-specific recommendations

The preceding sections converge on three findings: clinical efficacy is indication dependent, the safety profile is favourable and largely indication independent, and stewardship requires a clear distinction between prophylactic and adjunctive-therapeutic use. A blanket recommendation is not supported by the current evidence. Table 2 translates this into a two-tier matrix separating indications in which topical vancomycin may be considered individually from those in which it should not be used. The instruction to consider individually is conditional rather than discretionary, and it is worth making the conditions explicit. Case-by-case prophylactic use is defensible only where a predominantly gram-positive risk coincides with one or more recognised risk-amplifying factors: clinically relevant immunosuppression and diabetes mellitus in particular – the best-supported single indication, with reductions in surgical site infection of roughly three-quarters and in deep infection of approximately four-fifths reported in diabetic foot-and-ankle surgery (Wukich et al., 2015); an operatively treated high-risk fracture as defined in the VANCO trial (open fracture, compartment syndrome, staged fixation, or NNIS risk index of two or more) rather than fracture grade alone; a prior periprosthetic joint infection or deep SSI at the operative site; documented staphylococcal colonisation or a high local gram-positive bioburden; a high local prevalence of gram-positive SSI on institutional surveillance; or a high implant burden with substantial dead space. The mirror image defines where use is not justified – the standard-risk, clean, primary-implant scenario and any field carrying substantial gram-negative or polymicrobial risk (the lower lumbar and sacral region, the pelvis, contaminated wounds) – for the elementary reason that vancomycin powder provides gram-positive cover only and is no substitute for guideline-concordant systemic prophylaxis. These tiers rest on uneven evidence: the high-risk fracture indication on randomised post hoc data and the remaining “consider individually” indications on consensus framed by limited or heterogeneous data. The matrix is accordingly an aid to judgement and not a formal evidence grade.

**Table 2:** Indication-specific recommendation matrix for intrawound vancomycin powder in orthopaedic surgery. Recommendation tiers are distinguished typographically rather than by colour: bold denotes indications that may be considered individually and bold italic those that are not recommended. The matrix is an aid to clinical judgement and does not constitute a formal GRADE assessment.

Indication	Recommendation	Dose	Application plane	Rationale
Revision arthroplasty for PJI (septic revision)	Consider individually	1000–2000 mg	Subfascial/ implant directed	Adjunctive-therapeutic rationale, Liao et al. (2022) supportive, embedded in comprehensive infection management
Chronic osteomyelitis/infected nonunion	Consider individually	1000–2000 mg	Into defect cavity	Pharmacodynamic plausibility, limited randomised evidence, individualised decision within infection team
High-risk fracture surgery (tibial plateau, pilon, open fractures)	Consider individually	1000 mg	Over implant/split	VANCO trial: significant post hoc reduction in gram-positive deep infections (O'Toole et al., 2021)
Instrumented spinal surgery (high-risk profile)	Consider individually	1000 mg	Suprafascial or split	Cohort data supportive, RCT evidence heterogeneous, guidelines do not currently issue a general recommendation
Primary total hip or knee arthroplasty (including high risk)	Not recommended	–	–	VPIP: no benefit, NNT ≈500, stewardship and off-label concerns outweigh any residual effect
Primary arthroplasty, standard risk, elective procedures without implants	Not recommended	–	–	No evidence of benefit, stewardship concerns outweigh speculative gain

NNT, number needed to treat; PJI, periprosthetic joint infection; RCT, randomised controlled trial.

Translating this framework into clinical practice requires institutional standardisation – more so because real-world protocols vary widely in dose, application plane, drain management, and antiseptic co-interventions, a heterogeneity that confounds the existing literature and argues for written rather than informal practice. Five elements are essential: a written local standard operating procedure that is agreed on between orthopaedic surgery, hospital hygiene, infectious diseases, and pharmacy; explicit discussion of the off-label status during preoperative consent; structured documentation of dose, application plane, and indication; periodic surveillance of SSI rates and pathogen profiles; and continuous education on the evidence base and stewardship principles.

## 9 Open questions and future directions

Several priorities emerge for future research. First, SSI and PJI definitions should be aligned to MSIS/ICM and CDC criteria across studies to improve comparability. Second, drain management should be systematically recorded because it directly influences the local elution curve and has been inconsistently reported across existing trials. Third, pharmacokinetic measurement of local and systemic concentrations at defined intervals would clarify the decay kinetics of Fig. 1 for specific dose regimens. Fourth, a pragmatic head-to-head randomised comparison of subfascial versus suprafascial application with concurrent pharmacokinetic profiling would close the most important remaining evidence gap identified in Sect. 5. Fifth, and in our view most consequential, the long-term ecological impact of widespread topical glycopeptide exposure – on local resistance patterns, on the prevalence of vancomycin-intermediate and vancomycin-resistant organisms, and on the breakthrough-pathogen spectrum, including any downstream escalation to broad-spectrum systemic therapy – remains genuinely uncertain and can be resolved only by sustained, indication-stratified microbiological surveillance at population scale (Table 2).

This review carries the limitations inherent to a narrative synthesis, which we prefer to state plainly rather than minimise. No formal risk-of-bias appraisal (for example RoB 2 or ROBINS-I) and no quantitative pooling were undertaken; the recommendations derive from expert consensus framed by the evidence rather than from a pre-specified, reproducible methodology. The focus on English-language and German guideline sources may underrepresent evidence from other regions, and the interpretation of pathogen shift and of sterile-wound complications in particular rests on a limited number of heterogeneous studies. Readers should therefore consult the cited primary trials, meta-analyses, and guidelines when applying the framework to specific clinical contexts.

## 10 Conclusions

Intrawound vancomycin powder has passed through a period of rapid uptake and now enters a phase of more differentiated assessment. A decade of predominantly positive retrospective data established the technique as an adjunctive prophylactic measure; the two major randomised trials of the current decade have added important prospective evidence. The VPIP trial is the principal recent contribution: in 1901 high-risk patients undergoing primary hip or knee arthroplasty, it demonstrated no benefit of topical vancomycin, dilute povidone-iodine, or their combination over saline, and the biostatistical projection to 80 000 patients indicates that a clinically relevant residual effect in this indication is improbable.

The recommendation matrix proposed here (Table 2) replaces a uniform policy with a framework that differentiates by indication, consistent with the broader international guideline landscape, and it allows us to state our own position plainly. For routine prophylaxis in clean primary hip or knee arthroplasty and in standard-risk elective surgery, we hold that topical vancomycin should be abandoned: it confers no demonstrable benefit, its number needed to treat is prohibitive, and its off-label status and stewardship cost are unjustified in the absence of efficacy – a stance that is also our own institutional practice. We do not, however, advocate abandoning it in the adjunctive-therapeutic setting. It may still be considered individually in high-risk fracture surgery on the basis of the VANCO trial, in instrumented spinal surgery within the heterogeneous evidence acknowledged by multiple guideline bodies, and above all in the septic revision setting, where its adjunctive-therapeutic rationale is pharmacodynamically coherent with the goals of infection management. Across all indications, 1000 mg remains the pragmatic standard dose; sub-inhibitory dosing is biologically hazardous (Fig. 1), and escalation above 2000 mg is justified only in revision scenarios. The application plane should be chosen according to anatomical and pharmacodynamic logic rather than habit, and the off-label character and clinical importance of the agent argue for restrictive indication, structured documentation, and institutional standardisation.

The clinical question is therefore not whether topical vancomycin “works”, but in which specific contexts its use can be justified at the intersection of efficacy, safety, and antimicrobial stewardship. The matrix proposed here is an attempt to frame that question in a way that is usable at the bedside.

## Data Availability

No code was generated and no primary data were produced for this review; all data discussed are available in the cited primary publications.
